# Becoming More Rugged and Better Resourced: The R2 Resilience Program’s© Psychosocial Approach to Thriving

**DOI:** 10.3389/fpsyg.2021.745283

**Published:** 2021-12-10

**Authors:** Michael Ungar, Philip Jefferies

**Affiliations:** Faculty of Health, Resilience Research Centre, Dalhousie University, Halifax, NS, Canada

**Keywords:** resilience, well-being, multisystemic, resources, positive adaptation, positive development, coping, stress

## Abstract

The past decade has seen growing interest in interventions that build resilience as a complementary practice to trauma-informed care. From school-based programs focused on self-regulation and academic success to programs that support the well-being of disadvantaged populations or healthcare workers at risk of burnout, the concept of resilience is being used most commonly for programming that builds the capacity of individuals to adapt under conditions of adversity. Critiques have raised concerns that resilience-promoting programs demonstrate bias toward changing individual-level factors such as cognitions (e.g., mindfulness and grit), behavior (e.g., expressing gratitude and changing personal routines), or attachments (e.g., feeling secure in relationships) which help people adapt to socially toxic situations without changing access to the resources they require to overcome exposure to adverse psychosocial factors. This trend belies advances to the theory of resilience which support a more contextualized, multisystemic understanding of how external protective factors (resources) enhance individual qualities (ruggedness) and vice versa. Building on a multisystemic description of resilience, the R2 Resilience Program© was developed and piloted with six different populations ranging from clients of urban social services to workers in a long-term care facility, managers in the health care sector, staff of a Fortune 500 corporation, students in a primary to grade 12 school, and adult volunteers affiliated with an international NGO. Focused on building both individual ruggedness and enhancing people’s resources (the two Rs), the program provides contextualized content for each population by selecting from 52 resilience promoting factors with a strong evidence base to create training curricula that enhance the personal qualities and social, physical, and institutional resources most likely to support resilience. This paper reviews the justification for a multisystemic approach to designing resilience interventions and then explains the process of implementation of the R2 program. Preliminary findings are reported, which suggest the program is experienced as effective, with evaluations ongoing.

## Introduction: Background and Rationale

There are a growing number of programs to build resilience designed to complement trauma-informed interventions which have a narrower focus on alleviating symptoms after exposure to stress or adversity. This focus on resilience shifts attention from the factors that cause mental illness (at the individual level) or family, community, or institutional dysfunction (at a systemic level) to how people survive and thrive despite the challenges they experience.

Different conceptualizations of resilience over the years have influenced programs designed to improve coping capacity. Early perspectives likened resilience to a largely static, dispositional personality traits which were thought to explain why some individuals emerged relatively unscathed from disadvantage or trauma (Anthony and Cohler, 1987; Hu et al., 2015; Galatzer-Levy et al., 2018). Such perspectives could do little, however, to inform programming as the premise was that latent capacities needed early and sustained nurturing rather than later remediation. More recently, however, attention has shifted to the growth in people’s patterns of functioning over time despite a “bad start,” with individual resilience seen as malleable and a set of capacities that can be increased or trained (Masten, 2001; Luthans, 2002). This resulted in many resilience promoting initiatives focused on individual cognitions or behaviors, as evidenced by the popularity of training in self-regulation and positive thinking. Further research into the factors that contribute to such outcomes has revealed the complex interplay of person and environmental factors which have supported better accounts of resilience as a process of interaction. This perspective supports the view that positive developmental trajectories are possible when individuals have the personal, social, and physical resources they need for optimal development even when early life experiences may compromise a person’s realization of their potential (Hambrick et al., 2019). A multisystemic model of resilience such as this highlights the capacity of biopsychosocial and social-ecological systems (which provide these resources) to support internal and external conditions for well-being while enhancing the quality of life for different populations, in particular those impacted by structural disadvantage or conditions that threaten personal development (Smeeth et al., 2021; Ungar, 2021). Understood this way, resilience is the process whereby individuals navigate to the resources they need to function optimally, as well as the ability of individuals to negotiate for resources to be provided in contextually and culturally meaningful ways (Ungar, 2011). These dual processes of navigation and negotiation explain why individual qualities like grit, optimism, and self-regulation can only produce positive outcomes if social and physical ecologies provide opportunities for people to develop and apply their strengths.

Programs that emphasize individual change may produce short-term benefits, but adaptations are likely to decrease their impact on well-being over time unless an individual’s environment is also transformed (Prilleltensky, 2014) in ways that facilitate optimal growth. A dual focus on both personal and environmental change should, therefore, be the basis for intervention. However, programming to build resilience delivered by mental health professionals has typically privileged work focused on individual change alone. Other professions such as social work, anthropology, and community development have their own bias toward social transformation. Rarely are both processes the focus of intervention at the same time.

In this paper, we explore the justification for a multisystemic approach to designing resilience interventions and then describe a novel resilience program that utilizes this approach. The program is outlined, principles and processes are described, and early evaluation findings are reported, which suggests the program is experienced as effective.

## The Case for Multisystemic Approaches to Resilience Interventions

The bifurcation of fields of practice into micro and macro systemic interventions is being challenged by the emerging science of multisystemic resilience (Ungar and Theron, 2020; Ungar, 2021). To illustrate with one example among many, there is evidence that exposing a child who is structurally disadvantaged by race or ability (and who is experiencing symptoms associated with PTSD following exposure to domestic violence) to mindfulness-based stress reduction techniques will be less effective unless efforts are made to ensure that exposure to the violence ends and new opportunities for attachment are made available by culturally competent caregivers (Lo et al., 2019). The child’s resilience is, therefore, a function of both their capacity to cope well under stress and the capacity of their social and physical environments to facilitate positive development. While individual *ruggedness* may be sufficient to support well-being under conditions of normal stress, the greater the barriers to functioning experienced by an individual, the more important *resources* become.

We term this multisystemic perspective of resilience “R2” (in recognition of the need to address both rugged qualities of individuals and their access to resources). In practice, individuals with more internal capacities (e.g., a positive future orientation, problem-solving skills, self-regulation, etc.) tend to be more likely to take advantage of opportunities for relationships and to exploit opportunities for financial or academic success (e.g., Baron, 2004; Broadbent, 2016; Bouchard et al., 2017), while individuals with better access to external resources (e.g., good quality services, meaningful employment, opportunities for affordable housing and education, family supports, a safe community, etc.) tend to be more optimistic and show higher levels of motivation to accomplish life tasks (Clarke et al., 2012; Thomson et al., 2015; Roksa and Kinsley, 2019). These positive feedback loops implicate multiple systems either sequentially or concurrently. Put simply, resilience is a dynamic process in which we interact with the world around us to become our best selves despite exposure to atypical stress or adversity.

**FIGURE 1:**
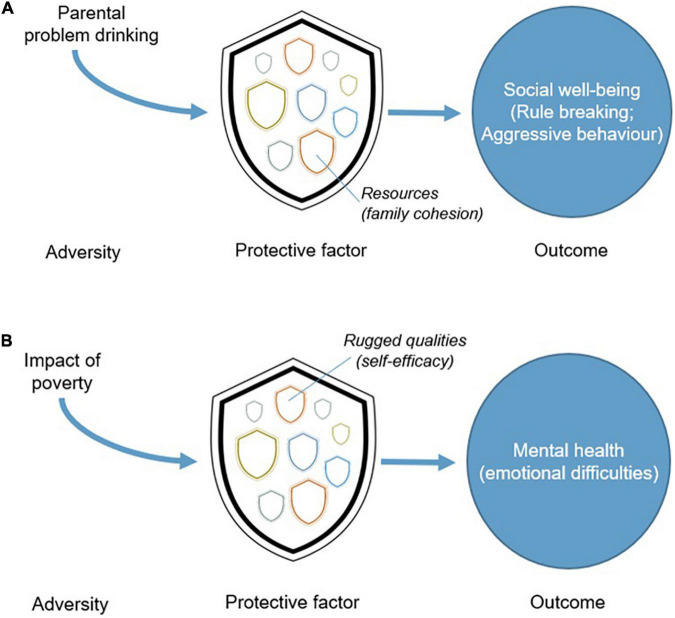
**(A,B)** Connections between adversity, protective factors, and outcomes.

Research supports this dual focus on ruggedness and resources. In a study of Danish schoolchildren, Meilstrup et al. (2016) found that self-efficacy was a protective factor that mediated the link between poverty and emotional difficulties (e.g., anxiety and depression). Specifically, children with greater self-efficacy had better mental health than their peers with low self-efficacy, despite their lower socio-economic status. In this case, the emphasis was on an individual quality mediating the impact of a structural constraint on development (poverty). In contrast, a study in the United States conducted by Finan et al. (2015) investigated the impact of parental problem drinking on children over time. They found that problem drinking was associated with later adolescent alcohol use, drug use, rule breaking, and aggressive behavior. However, they found that family cohesion (a social resource) impacted some of these outcomes, namely, rule breaking and aggressive behaviors. Commenting on their findings, Finan et al. (2015) called for programs to target ways to bring families closer together as a means of buffering the negative impact of a parent’s addiction. Studies like these suggest a pattern of systemic feedback, with the potential for any single personal quality or external resource to create a cascade of positive changes across multiple superordinate and subordinate systems. Together, both studies also illustrate three design principles that are common to effective interventions that promote resilience (Ungar, 2019). They must (1) identify the nature of the adversity individuals or groups of individuals experience, (2) match the right protective factor at the right system (or systems) level to that adversity, and (3) clearly articulate the desired behavioral outcome which is to be achieved (and which is reasonably likely given the nature of the protective process which is employed). Figure 1 illustrates this three-part model for resilience interventions applied to the two studies just mentioned.

## Pedagogical Framework

The R2 Resilience Program© was designed with principles of implementation science (Rycroft-Malone et al., 2013) to ensure that all three aspects of a successful intervention were accounted for in its design by matching the program content to local priorities of stakeholders. By doing so, the program avoids two common but fundamental flaws in programs that promote positive development. First, a multisystemic focus is less likely to unintentionally blame victims of oppressive conditions for their inability to change, compelling multiple systems to share responsibility for an individual’s successful transformation or, alternatively, their adaptation to stubbornly stable toxic life circumstances which impinge on psychosocial growth. For example, an employee who has been asked to achieve an unrealistically high sales quota or deal with sexual harassment in the workplace may benefit from techniques to self-regulate and perform work-related tasks but will also need support to address working conditions that make individual adaptations unsustainable or even, paradoxically, harmful if the toxicity of workplace relationships goes unchallenged. In this example, the locus of change rests with both the individual worker and the workplace as an institution.

The second design flaw common to interventions that promote resilience is that programming may target change at the wrong systemic level, producing little or no sustainable experience of well-being. For example, there is evidence that bullying among school-aged children is best addressed by changes to school policy (making the school a safe space), or by helping children who are bullied find same-age peers to befriend them (bullies tend to prey on children they perceive as socially isolated) rather than by empowering individual children to resist bullying on their own (Mishna et al., 2016). While social transformations are the better starting point, individual changes to cognitions (and other psychological interventions) for bullied children remain a necessary catalyst for a child to take advantage of a safer school environment and gain the confidence to engage socially with peers. On their own, however, efforts to improve individual coping strategies are likely to fail if the child’s environment remains unsafe.

## Resilience Programming and Positive Developmental Processes

There are many programs and interventions already in use that purport to build resilience. Some are aimed at the general public and target common adversities like stress (e.g., see Joyce et al., 2018) and burnout at work (Vanhove et al., 2016), while others are aimed at specific populations, such as healthcare professionals (Cleary et al., 2018), employees returning to work (Heathcote et al., 2019), and individuals managing chronic medical conditions such as hypertension and diabetes (Pesantes et al., 2015). These programs are diverse in the way that they are delivered, including single-day workshops, weekly sessions, or self-directed psychoeducation materials packaged in the form of online phone applications, printed manuals, or web-based tools. All can be completed at one’s own pace. Recent meta-analyses have found that such interventions are likely to have a small-to-moderate impact on enhancing resilience (Leppin et al., 2014; Joyce et al., 2018; Liu et al., 2020).

Existing resilience interventions also vary significantly in terms of the protective factors that they target. A scoping review now underway by the authors is finding that there are a broad range of protective factors that existing resilience interventions seek to improve, such as problem-solving skills, self-efficacy, and cognitive flexibility. Most of these programs, however, are overwhelmingly focused on modifying rugged factors, typically by changing individual behaviors or cognitions. A small number of interventions address resources external to the individual, with the most common focus being building and improving relationships with others (see also Chmitorz et al., 2018). Programs that address other resources critical to resilience such as improved access to social justice, health care, housing, or changes to how one is perceived in one’s community tend to be the focus of community development initiatives that work with people in group settings (Springgate et al., 2011). Very rarely do we find evidence of programs that explicitly target both individual coping strategies and strategies to create better resourced environments around individuals.

In developing the R2 intervention, we surveyed the vast resilience literature and then worked as a team of resilience scholars affiliated with the Resilience Research Centre to create a shortlist of rugged qualities and resources with sufficient evidence to show that enhancement of these factors would change an individual’s experience of resilience. While many of the factors we identified shared common elements, we were able to identify 26 relatively distinct rugged qualities and 26 critical resources (the symmetry is intentional to ensure equal attention is paid to both aspects of resilience) that have been well studied and which are known to be associated with resilience. These factors are applicable to multiple populations and conditions of adversity. A complete list of all 52 factors is included in Table 1. Together, these 26 rugged qualities and 26 resources present a list of potential protective factors that the R2 Resilience Program© draws on.

**TABLE 1:** The R2 Resilience Program’s© 52 resilience factors.

Rugged qualities	Resources
A powerful identity	A diverse community
Altruism	A supportive peer group
Communication skills	Access to mental and physical health care
Conscientiousness	Access to recreational facilities and outdoor spaces
Cooperation and help-seeking	Accountability/reasonable consequences for one’s actions/opportunities to fix one’s mistakes
Creativity	Advocacy if treated poorly
Critical thinking	Appropriate use of social media
Decision-making	Contact with extended family
Empathy	Contact with one’s elders
Flexibility	Cultural practices/family and community traditions
Goal-setting	Education/training
Gratitude	Equitable access to opportunities
Humor	“Good enough” parenting/caregiving
Meaning-making/spirituality	Housing, supports, and connectivity
Mindfulness and self-regulation	Meaningful employment
Morality	Mentors and mentoring
Motivation/perseverance	Opportunities to make decisions for oneself
Optimism/hope	Opportunities to use one’s talents
Physical activity	Orderly and regular routines
Positive emotions	Physical safety/public security
Problem-solving	Proper nutrition
Self-actualization	Protection from discrimination and respect for one’s rights
Self-care/compassion for self	Reasonable expectations for how one should behave
Self-efficacy	Relationships with others in one’s community
Self-esteem/confidence	Social efficacy and citizenship
Sleep hygiene	Transportation

## The R2 Resilience Program© Objectives: Creating a Contextually Responsive Multisystemic Intervention

The R2 Resilience Program© is a curriculum-based approach to enhancing the resilience of populations experiencing atypical stress or adversity. The program is contextualized to fit the specific needs of an organization, educational institution, or business setting. Ideally, every implementation of the R2 approach accomplishes the following:

• *Explores the general and specific risks faced by individuals in each work or service setting*. Whether these risks are related to individual factors like psychological trauma, institutional conditions like corporate restructuring, or a major event like a natural disaster, R2 responds to the issues that are most pressing locally. To tailor the intervention, R2 leaders meet with members of a senior management team and those responsible for the health and well-being of staff/students/clients to ensure the program is adapted to the specific challenges and opportunities people experience daily.
• *Identifies the range of resilience factors that are right for each setting*. Different organizations, institutions, and businesses need different protective factors to support their staff/students/clients.
• *Identifies the right audience for the program*. Content is created that focuses on the needs of individuals and groups seeking to build their resilience, as well as those holding positions of responsibility for others. The R2 curriculum can be adapted to meet the needs of individual staff, customers, and students, or adapted to include case material to support the work of organizational leaders.
• *Formats the delivery of the program to ensure it fits each organizational setting and the time and resources each has to build resilience*. R2 curriculum is intentionally designed to be turned into workshops and online resources. Delivery can vary from a series of short interactive seminars delivered face-to-face to day-long events, websites, and apps, depending on what each organization needs. The program can be offered in different mediums, such as face-to-face coaching, group workshops, or online, and can be facilitated by an R2 expert, an internally trained trainer, or self-directed by participants through direct access to learning materials. This flexibility is accomplished through the modular formatting of the content and repurposing curriculum as new applications are requested.
• *Makes the training materials easily accessible to ensure the program is sustainable*. Once an R2 program is developed, organizations continue to access these resources at a minimum cost. As the program grows, changes can be reflected in the materials that are shared.
• *Supports the design of an evaluation to measure outcomes*. Evaluations can range from brief and minimally intrusive to far larger, multisite longitudinal studies of outcomes depending on the capacity of each setting and available funding. In most cases, evaluation tools are built into the delivery of the R2 curriculum through pre- and post-tests and self-assessment exercises embedded in each module that explores a different resilience factor. In this way, areas of intended change are measured and data can be made easily accessible to end-users.

To date, R2 has been piloted with six different populations ranging from clients of urban social services, to workers in a long-term care facility, managers in the health care sector, staff of a Fortune 500 corporation, students in a primary to grade 12 school, and adult volunteers affiliated with an international NGO. Focused on building both individual ruggedness and enhancing people’s resources (the two Rs), the program provides contextualized content for each population. Specific goals for the intervention include: engaging participants in meaningful conversations about the many (multisystemic) factors that nurture and sustain resilience; providing practical strategies for improving individual ruggedness and access to, and use of, social, built, and natural resources; and when required, adding to the competencies of professionals tasked with enhancing the resilience of individuals and communities. To create and then implement the content to fulfill these goals, the program proceeds through four phases of intervention; contextualization, offsite program development, implementation, and evaluation.

### Phase 1: Contextualization

A Delphi process (Dalkey, 1968) prior to piloting ensures the program is theoretically sound but matched to the risk profiles of participants. As no single program could cover all 52 factors, nor would all 52 factors be relevant to every population experiencing atypical stress or adversity, the first phase of the R2 Program is to work with senior management and representatives (clients/staff/residents) of each setting where the program is to be implemented to identify: (1) the most relevant of the 52 resilience factors; (2) the format the programming will take (this includes the amount of time the setting can devote to the program implementation and whether content will be delivered in person, through online synchronous or asynchronous webinars, or whether training will be direct with people associated with the setting or delivered through a train-the-trainer model of implementation); and (3) the pedagogical approach most likely to fit the needs of program participants (lecture, workshops, experiential exercises, discussion groups, homework assignments, personal reflection exercises, etc.).

To conduct the Delphi survey, members of an organization that are best placed to help determine which of the factors would form a priority R2 program are invited to rank order the 52 resilience factors. Respondents may include a mixture of senior management, program administrators, or even recipients of the forthcoming program (if the target group is not the whole of the organization) who may be the “experts by experience” (Iqbal and Pipon-Young, 2009). These individuals form the panel involved in the Delphi method. Involving individuals in this way is not only important for drawing on their knowledge and expertise, but can enhance overall engagement, perceptions of ownership, and acceptance of outcomes (McKenna, 1994; Keeney et al., 2001). The Delphi method, originally developed in the 1950s by the RAND Corporation, is now widely used in the social sciences as a means of reducing a number of possible options and arriving at consensus among experts (Vernon, 2013). The traditional approach involved face-to-face interactions but has since been adapted into more flexible “e-Delphi” forms where online surveys and emails can be used to gather responses asynchronously from geographically dispersed individuals (Thangaratinam and Redman, 2005). When developing an R2 program with organizations, we offer an e-Delphi approach but recommend that the Delphi be included as part of introductory meetings with an organization to expedite the process. These meetings, which can be conducted face-to-face or virtually, familiarize participants with the R2 approach and the importance of addressing both rugged qualities and resources in processes of resilience building. Each of the R2 factors is briefly described and participants are then invited to access a survey tool where they can anonymously rank each of the rugged qualities and then each of the resource qualities. When ranking the factors, participants are encouraged to consider whether they believe the factor is:

• Something that individuals are in need of;
• Something not being adequately addressed elsewhere by the organization, institution, or business;
• Something that individuals will find important (something they may enjoy, appreciate, or respond positively to);
• Something that a program could realistically improve.

Participants are encouraged to distinguish resilience factors that are important and already being addressed by their organization, institution, or business, and those that they would like to see new programming address. In this way, R2 extends the capacity of an organization for resilience rather than simply replicating effective programming already in place.

If this process is conducted during a live session (a virtual or face-to-face meeting), responses are automatically pooled and are then presented back to the group immediately. The group is reminded that these are only results from a first round and that the highest ranked choices are a potential shortlist for the program. Time is then allocated for discussion, where participants are invited to discuss the outcome of the first round, which may involve reflecting on the appropriateness of those items currently at the top of the lists or arguing for the importance of factors that were not very highly ranked. We also contribute to these discussions where relevant; for instance, noting factors that have been successfully included in similar contexts.

A second round of ranking then takes place. If the outcome is substantially different from the first round, further discussion takes place to review these changes. Two to three rounds are normally required to reach a general consensus (Stone Fish and Busby, 2005). Once achieved, the group is informed that a priority list has been identified and an equal number of the top rugged qualities and resources are then selected. These are then developed into curriculum based on the logistical and pedagogical needs of a particular setting.

While the Delphi process helps to ensure that the most relevant factors related to resilience are the focus of programming, results may be skewed by who was selected to complete the ranking with those most in need of resilience training but less motivated to participate silenced by the process. While consensus can be achieved, this does not necessarily mean that Delphi conclusions reflect consensus across an entire organization. Results should therefore be considered cautiously and in light of who was selected as an expert and whether additional areas of expertise are required to refine results.

### Phase 2: Offsite Program Development

The R2 team uses the information gathered during Phase 1 to develop a tailored version of the R2 program that fits the need of the host organization, institution, or business. The time required to develop the program will vary depending on the extent of the adaptation needed but typically can be accomplished within 4–6 weeks. An evaluation plan and evaluation materials are also developed at the same time. The short timeframe helps to maintain the momentum of the work already done. Each module contains common elements. These include:

1. An introduction to the module and what participants can expect to learn. The material, while theoretically sound, is meant to be accessible and brief, with a heavy emphasis on the activities R2 Resilience Program© participants can do to integrate new resilience-enabling patterns of behavior into their lives.
2. A brief overview of the resilience factor, as well as the typical risk factors which threaten individuals and make the factor more or less important in different contexts. This section of the module includes a plain language scientific summary that reviews the available evidence for the effectiveness of each resilience factor.
3. Validated measures from the literature that can be used to help participants reflect on the impact each resilience factor may have on their lives. Where non-validated measures are recommended (e.g., when validated measures do not exist), anecdotal evidence of their performance as assessment tools is provided.
4. A series of sensitizing questions that can be used in place of standardized measures to facilitate self-reflection and group discussion of the module’s content. These sensitizing questions are starting prompts to help participants consider the wide range of implications each resilience factor has on their lives and the lives of others across their organization, institution, or business.
5. Case studies that illustrate how the module’s content reflects actual stories of successful recovery, adaptation and transformation in each organization, institution, or business. These case illustrations are developed through an appreciative inquiry process in which those attached to a specific setting are asked to tell stories of past successes developing resilience and to identify the constellation of personal qualities and external supports that they received which made their success possible.
6. Sample lesson plans for facilitating workshop content specific to each resilience factor. Where available, visual materials like PowerPoint slides and links to online videos may be included. These lesson plans are provided in the format requested by the host organization, institution, or business but are usually malleable so that the program can be easily adapted to different groups of participants with different learning needs or the amount of time they have to devote to the training.
7. The expected outcomes from completion of the module. Participants are provided with a summary of the changes they can expect from their use of the module with specific details about potential benefits to mental health and one’s ability to cope in social contexts that produce stress. Specific learning objectives are also developed for each setting, which are tailored to the selected protective factors required for the program. For example, a program involving self-efficacy will include learning objectives related to conceptual understanding of the concept and application of activities tailored for the selected setting that improve self-efficacy.

### Phase 3: Implementation of the Contextualized Program

With the modules developed, the R2 team returns to the host setting, this time presenting a general introduction to R2 to as many staff and other stakeholders as possible. The R2 experts then either (a) work with a small group of individuals to conduct a trial of the program (refinement of the materials is then done offsite and a revised version of the program returned to the organization, institution, or business for implementation) or (b) train a group of individuals within the organization or business who will be the R2 trainers, supporting them after the training is completed and implementation of the R2 curriculum begins.

### Phase 4: Evaluation

Whenever possible, an evaluation of outcomes is conducted by either R2 experts or by those with this expertise already employed by the organization, institution, or business. Typically, evaluations occur at regular intervals (e.g., on the first day of training, and 3 and 6 months after the training ends). Evaluation staff at the Resilience Research Centre are available to help compile and analyze the data, produce a report on the findings, and make recommendations for further tailored implementation of the R2 program.

## Contextualized vs. Standardized Versions of the R2 Resilience Program©

By using these four phases, R2 can be offered as a tailored intervention that is contextually and culturally relevant. Over time, however, and to make access to the program cost-effective, several versions of the program intended for similar populations are being aggregated into “standardized” versions of R2. Implementation of standardized versions still includes a period of contextualization where case examples are adapted and pedagogical techniques are matched to the needs of each setting’s participants, though the selection of the factors and much of the content of each module remains constant.

**FIGURE 2:**
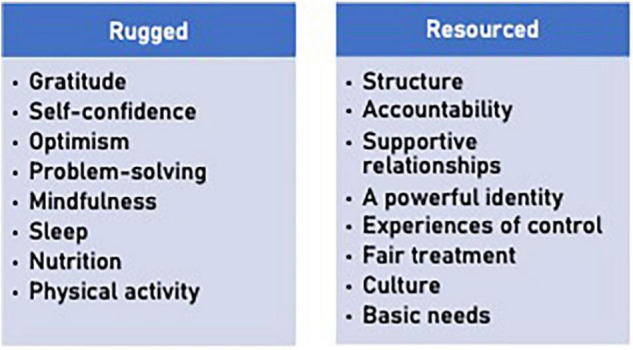
Example of intervention selection of rugged qualities and resources. The factors selected in this Delphi process were selected from an earlier version of Table 1. The wording of some items has changed slightly over time.

To illustrate this process, a version of the R2 program was developed for use by a Fortune 500 company conducting a corporate social responsibility campaign to enhance the resilience of young adults with social anxiety. Through a Delphi process with stakeholders, 16 items were selected during Phase 1 of implementation (see Figure 2).

**FIGURE 3:**
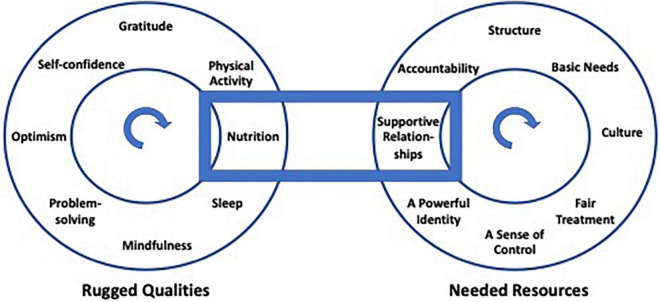
Interaction between rugged qualities and resources.

In developing the modules to explain each factor, efforts were made to demonstrate the interconnections between them. Reflecting the science of resilience, factors, and processes associated with resilience interact such that every rugged feature depends on resources to facilitate growth, just as every resource requires the development of individual qualities to cope with atypical stressors. This dynamic model (see Figure 3) ensures the enhancement of both ruggedness and resourcefulness at the same time. To illustrate, participants in this version of R2 were shown that a change in nutrition and eating habits (an individual quality) is partially dependent on the relationships we have with others, our access to healthy food and income, as well as cultural norms which shape what we eat, and when and how food is prepared. Likewise, experiences of personal control that we experience in our workplaces or families influence self-confidence and a sense of optimism for a future that individuals exercise some control over. These patterns were shown by graphicly representing the 16 factors as points on two dials that each rotate, aligning factors in different combinations.

## Discussion

The R2 Resilience Program© is an evidence-informed approach to building resilience that purposefully provides participants with the opportunity to tailor the content. Variation in the number of factors chosen during each application and the factors that are chosen suggests that the goal of matching the program to different contexts and cultures has been effective. While results are still being assessed, initial evaluation data suggest the program is helping participants identify strategies that are useful when dealing with exposure to significant threats to their mental well-being. For example, 65 volunteers with an international service club participated in a trial version of R2 in April 2021. Between pre- and post-intervention, participants experienced a reduction in anxiety and depression symptoms, as well as perceived stress. Volunteers’ anxiety symptoms, assessed using the seven-item Generalized Anxiety Disorder (GAD-7) scale (Spitzer et al., 2006) decreased substantially from an average of 12.2 at Time 1 (SD = 5.4) to an average of 9 at Time 2 (SD = 2.1). Similarly, the eight-item Patient Health Questionnaire (PHQ-8) depression scale (Kroenke et al., 2009) was used to measure changes in volunteers’ depression symptoms, with scores decreasing slightly from 11.9 at Time 1 (SD = 3.2) to 10.4 at Time 2 (SD = 1.6). Following their participation in the R2 pilot program, volunteers also experienced a decrease in perceived stress as measured by the Perceived Stress Scale (PSS) (Cohen, 1988). Overall, average scores on the 10-item PSS dropped from 22.7 at Time 1 (SD = 6.8) to 19.3 at Time 2 (SD = 4.7). With regard to resilience, scores on the Brief Resilience Scale (Smith et al., 2008) remained stable between pre- and post-intervention assessments (*M* = 25.1, SD = 3.1 and *M* = 25.4, SD = 3.5, respectively). These results should be interpreted in light of the challenges brought about by the COVID-19 pandemic and the associated stay-at-home orders and restrictions people experienced. Although the resilience of the volunteers did not increase following their participation in the R2 program, stability in resilience levels at this time of great psychosocial distress should be interpreted as a positive outcome. Finally, when volunteers were asked if, overall, the R2 pilot program sessions were helpful in showing them how to build their personal resilience, 80% of those surveyed said the sessions were indeed helpful or very helpful. Similarly, 80% found the sessions helpful for generating ideas to improve their community’s resilience, and 86.7% stated that the sessions and the materials that were provided were easy or very easy to understand and follow. Among the components of the R2 pilot program that volunteers enjoyed the most, they listed the breakout sessions that gave participants a chance to discuss resilience issues in a group setting with other participants and the integration of case examples. Results from other trials are showing similar trends in outcomes.

Pedagogically, the approach to building resilience supported by R2 provides program participants with the opportunity to select from a menu of options the factors at multiple systemic levels most likely to produce positive outcomes. This approach, like other effective resilience interventions that adapt across cultures (see Cesana et al., 2018) shows promise of helping mental health professionals discover locally relevant ways of supporting individuals of all ages and abilities across a plurality of cultures and contexts to enhance both individual and social-ecological factors associated with successful adaptation and transformation under stress.

## Constraints

While the content of R2 adapts when implemented in different settings, this strength in contextual relevance can undermine the program’s fidelity, requiring facilitators to ensure each application adheres to a set of principles (e.g., equal focus on ruggedness and resources) while tolerating unique expressions of the program content. Differences in the length of the program and variations in pedagogical techniques also raise questions about the dosage effect of the program and whether participants are choosing to design the program in the most efficacious way. We expect that with more trials and more evaluations, we will be able to guide organizations, institutions, and businesses better with regard to how they offer the R2 Resilience Program© in each setting.

Despite this challenge, resilience programs (like R2) are most likely to be effective when individuals are given access to the resources they require and when these resources are provided in ways that are contextually and culturally meaningful. This means, for example, that persons in positions of power should be involved in programs to help facilitate access to important resources. For instance, if safety and security are a concern in a given organizational context, then in addition to person-centered activities such as encouraging recognition of safe or unsafe practices and articulating safety needs, senior staff need to be engaged in creating the mechanisms for employees to access the supports required to remain safe. Therefore, given an understanding of resilience as process instead of trait, programs like R2 depend on both individual action and the actions of other co-occurring systems to create an optimal context for personal and collective development.

## Data Availability Statement

The original contributions presented in the study are included in the article/supplementary material; further inquiries can be directed to the corresponding author.

## Ethics Statement

Ethical review and approval were not required for the study on human participants in accordance with the local legislation and institutional requirements. The ethics committee waived the requirement of written informed consent for participation.

## Author Contributions

MU led the development of the R2 program and drafted 70% of the manuscript. PJ contributed significantly to the development of the R2 curriculum and the implementation of the program and wrote the remainder of the manuscript. Both authors contributed to the article and approved the submitted version.

## Conflict of Interest

The authors declare that the research was conducted in the absence of any commercial or financial relationships that could be construed as a potential conflict of interest.

## Publisher’s Note

All claims expressed in this article are solely those of the authors and do not necessarily represent those of their affiliated organizations, or those of the publisher, the editors and the reviewers. Any product that may be evaluated in this article, or claim that may be made by its manufacturer, is not guaranteed or endorsed by the publisher.

## References

[B1] Anthony E. J. Cohler B. J. (eds) (1987). *The Invulnerable Child.* New York: Guilford Press.

[B2] Baron R. A. (2004). “Opportunity recognition: insights from a cognitive perspective,” in *Opportunity Identification and Entrepreneurial Behavior*, ed. Butler J. E. (Charlotte: Information Age Publishing), 47–73.

[B3] Bouchard L. C. Carver C. S. Mens M. G. Scheier M. F. (2017). “Optimism, Health, and Well-Being,” in *Positive Psychology*, ed. Dunn D. S. (Milton Park: Routledge), 112–130.

[B4] Broadbent J. (2016). Academic success is about self-efficacy rather than frequency of use of the learning management system. *Australas. J. Educ. Technol.* 32 38–49. 10.14742/ajet.2634

[B5] Cesana M. L. Giordano F. Boerchi D. Rivolta M. Castelli C. (2018). Drawing to reconstruct: pilot study on acknowledging prisoners’ internal and external resources in a penitentiary institution. *World Futures* 74 392–411. 10.1080/02604027.2018.1445913

[B6] Chmitorz A. Kunzler A. Helmreich I. Tüscher O. Kalisch R. Kubiak T. (2018). Intervention studies to foster resilience – A systematic review and proposal for a resilience framework in future intervention studies. *Clin. Psychol. Rev.* 59 78–100. 10.1016/j.cpr.2017.11.002 29167029

[B7] Clarke P. J. Ailshire J. A. House J. S. Morenoff J. D. King K. Melendez R. (2012). Cognitive function in the community setting: the neighbourhood as a source of ‘cognitive reserve’?. *J. Epidemiol. Community Health* 66 730–736. 10.1136/jech.2010.128116 21515547 PMC3387518

[B8] Cleary M. Kornhaber R. Thapa D. K. West S. Visentin D. (2018). The effectiveness of interventions to improve resilience among health professionals: a systematic review. *Nurs. Educ. Today* 71 247–263. 10.1016/j.nedt.2018.10.002 30342300

[B9] Cohen S. (1988). “Perceived stress in a probability sample of the United States,” in *The Social Psychology Of Health*, eds Spacapan S. Oskamp S. (Thousand Oaks: Sage Publications Inc), 31–67.

[B10] Dalkey N. C. (1968). *The Delphi Method: An Experimental Study of Group Opinion.* California: RAND.

[B11] Finan L. J. Schulz J. Gordon M. S. Ohannessian C. M. (2015). Parental problem drinking and adolescent externalizing behaviors: the mediating role of family functioning. *J. Adolesc.* 43 100–110. 10.1016/j.adolescence.2015.05.001 26073673 PMC4516616

[B12] Galatzer-Levy I. R. Huang S. H. Bonanno G. A. (2018). Trajectories of resilience and dysfunction following potential trauma: a review and statistical evaluation. *Clin. Psychol. Rev.* 63 41–55. 10.1016/j.cpr.2018.05.008 29902711

[B13] Hambrick E. P. Brawner T. W. Perry B. D. Brandt K. Hofmeister C. Collins J. O. (2019). Beyond the ACE score: examining relationships between timing of developmental adversity, relational health and developmental outcomes in children. *Arch. Psychiatr. Nurs.* 33 238–247. 10.1016/j.apnu.2018.11.001 31227076

[B14] Heathcote K. Wullschleger M. Sun J. (2019). The effectiveness of multi-dimensional resilience rehabilitation programs after traumatic physical injuries: a systematic review and meta-analysis. *Disabil. Rehabil.* 41 2865–2880. 10.1080/09638288.2018.1479780 29933700

[B15] Hu T. Zhang D. Wang J. (2015). A meta-analysis of the trait resilience and mental health. *Pers. Individ. Differ.* 76 18–27. 10.1016/j.paid.2014.11.039

[B16] Iqbal S. Pipon-Young L. (2009). The Delphi method | The Psychologist. *Psychologist* 2009 598–601.

[B17] Joyce S. Shand F. Tighe J. Laurent S. J. Bryant R. A. Harvey S. B. (2018). Road to resilience: a systematic review and meta-analysis of resilience training programmes and interventions. *BMJ Open* 8:e017858. 10.1136/bmjopen-2017-017858 29903782 PMC6009510

[B18] Keeney S. Hasson F. McKenna H. P. (2001). A critical review of the Delphi technique as a research methodology for nursing. *Int. J. Nurs. Stud.* 38 195–200. 10.1016/S0020-7489(00)00044-4 11223060

[B19] Kroenke K. Strine T. W. Spitzer R. L. Williams J. B. W. Berry J. T. Mokdad A. H. (2009). The PHQ-8 as a measure of current depression in the general population. *J. Affect. Disord.* 114 163–173. 10.1016/j.jad.2008.06.026 18752852

[B20] Leppin A. L. Bora P. R. Tilburt J. C. Gionfriddo M. R. Zeballos-Palacios C. Dulohery M. M. (2014). The Efficacy of Resiliency Training Programs: a systematic review and meta-analysis of randomized trials. *PLoS One* 9:e111420. 10.1371/journal.pone.0111420 25347713 PMC4210242

[B21] Liu J. J. W. Ein N. Gervasio J. Battaion M. Reed M. Vickers K. (2020). Comprehensive meta-analysis of resilience interventions. *Clin. Psychol. Rev.* 82:101919. 10.1016/j.cpr.2020.101919 33045528

[B22] Lo H. H. M. Wong J. Y. H. Wong S. W. L. Wong S. Y. S. Choi C. W. Ho R. T. H. (2019). Applying Mindfulness to Benefit Economically Disadvantaged Families: a Randomized Controlled Trial. *Res. Soc. Work Pract.* 29 753–765. 10.1177/1049731518817142

[B23] Luthans F. (2002). The need for and meaning of positive organizational behavior. *J. Organ. Behav.* 23 695–706. 10.1002/job.165

[B24] Masten A. S. (2001). Ordinary magic. Resilience processes in development. *Am. Psychol.* 56 227–238. 11315249 10.1037//0003-066x.56.3.227

[B25] McKenna H. P. (1994). The Delphi technique: a worthwhile research approach for nursing?. *J. Adv. Nurs.* 19 1221–1225. 10.1111/j.1365-2648.1994.tb01207.x 7930104

[B26] Meilstrup C. Thygesen L. C. Nielsen L. Koushede V. Cross D. Holstein B. E. (2016). Does self-efficacy mediate the association between socioeconomic background and emotional symptoms among schoolchildren?. *Int. J. Public Health* 61 505–512. 10.1007/s00038-016-0790-3 26841894

[B27] Mishna F. Khoury-Kassabri M. Schwan K. Wiener J. Craig W. Beran T. (2016). The contribution of social support to children and adolescents’ self-perception: the mediating role of bullying victimization. *Child. Youth Serv. Rev.* 63 120–127. 10.1016/j.childyouth.2016.02.013

[B28] Pesantes M. A. Lazo-Porras M. Abu Dabrh A. M. Ávila-Ramírez J. R. Caycho M. Villamonte G. Y. (2015). Resilience in Vulnerable Populations With Type 2 Diabetes Mellitus and Hypertension: a Systematic Review and Meta-analysis. *Can. J. Cardiol.* 31 1180–1188. 10.1016/j.cjca.2015.06.003 26239007 PMC4556590

[B29] Prilleltensky I. (2014). Justice and Human Development. *Int. J. Educ. Psychol.* 3 287–305. 10.4471/ijep.2014.15

[B30] Roksa J. Kinsley P. (2019). The role of family support in facilitating academic success of low-income students. *Res. High. Educ.* 60 415–436. 10.1007/s11162-018-9517-z

[B31] Rycroft-Malone J. Seers K. Chandler J. Hawkes C. A. Crichton N. Allen C. (2013). The role of evidence, context, and facilitation in an implementation trial: implications for the development of the PARIHS framework. *Implement. Sci.* 8:28. 10.1186/1748-5908-8-28 23497438 PMC3636004

[B32] Smeeth D. Beck S. Karam E. G. Pluess M. (2021). The role of epigenetics in psychological resilience. *Lancet Psychiatry* 8 620–629. 10.1016/S2215-0366(20)30515-0 33915083 PMC9561637

[B33] Smith B. W. Dalen J. Wiggins K. Tooley E. Christopher P. Bernard J. (2008). The brief resilience scale: assessing the ability to bounce back. *Int. J. Behav. Med.* 15 194–200. 10.1080/10705500802222972 18696313

[B34] Spitzer R. L. Kroenke K. Williams J. B. W. Löwe B. (2006). A Brief Measure for Assessing Generalized Anxiety Disorder: the GAD-7. *Arch. Intern. Med.* 166:1092. 10.1001/archinte.166.10.1092 16717171

[B35] Springgate B. F. Wennerstrom A. Meyers D. Iii C. E. A. Vannoy S. D. Bentham W. (2011). Building Community Resilience through Mental Health Infrastructure and Training in Post-Katrina New Orleans. *Ethn. Dis.* 21 S1–20-9. PMC3731130 22352077

[B36] Stone Fish L. Busby D. M. (2005). “The Delphi Method,” in *Research methods in family therapy, 2nd ed*, eds Sprenkle D. H. Piercy F. P. (New York: The Guilford Press), 238–253.

[B37] Thangaratinam S. Redman C. W. (2005). The Delphi technique. *Obstet. Gynaecol.* 7 120–125. 10.1576/toag.7.2.120.27071

[B38] Thomson K. C. Schonert-Reichl K. A. Oberle E. (2015). Optimism in early adolescence: relations to individual characteristics and ecological assets in families, schools, and neighborhoods. *J. Happiness Stud.* 16 889–913. 10.1007/s10902-014-9539-y

[B39] Ungar M. (2011). The social ecology of resilience: addressing contextual and cultural ambiguity of a nascent construct. *Am. J. Orthopsychiatry* 81 1–17. 10.1111/j.1939-0025.2010.01067.x 21219271

[B40] Ungar M. (2019). Designing resilience research: using multiple methods to investigate risk exposure, promotive and protective processes, and contextually relevant outcomes for children and youth. *Child Abuse Negl.* 96:104098. 10.1016/j.chiabu.2019.104098 31376582

[B41] Ungar M. (2021). *Multisystemic Resilience: Adaptation and Transformation in Contexts of Change.* Oxford: Oxford University Press.

[B42] Ungar M. Theron L. (2020). Resilience and mental health: how multisystemic processes contribute to positive outcomes. *Lancet Psychiatry* 7 441–448. 10.1016/S2215-0366(19)30434-1 31806473

[B43] Vanhove A. J. Herian M. N. Perez A. L. U. Harms P. D. Lester P. B. (2016). Can resilience be developed at work? A meta-analytic review of resilience-building programme effectiveness. *J. Occup. Organ. Psychol.* 89 278–307. 10.1111/joop.12123

[B44] Vernon W. (2013). The Delphi technique: a review. *Int. J. Ther. Rehabil*. 16 69–76. 10.12968/ijtr.2009.16.2.38892

